# Supplementary figures and images for: Enhancing reproducibility in single cell research with biocytometry: An inter-laboratory study
Source: PLoS One. 2024 Dec 9;19(12):e0314992. doi: 10.1371/journal.pone.0314992 (PMC11627387; doi:10.1371/journal.pone.0314992)

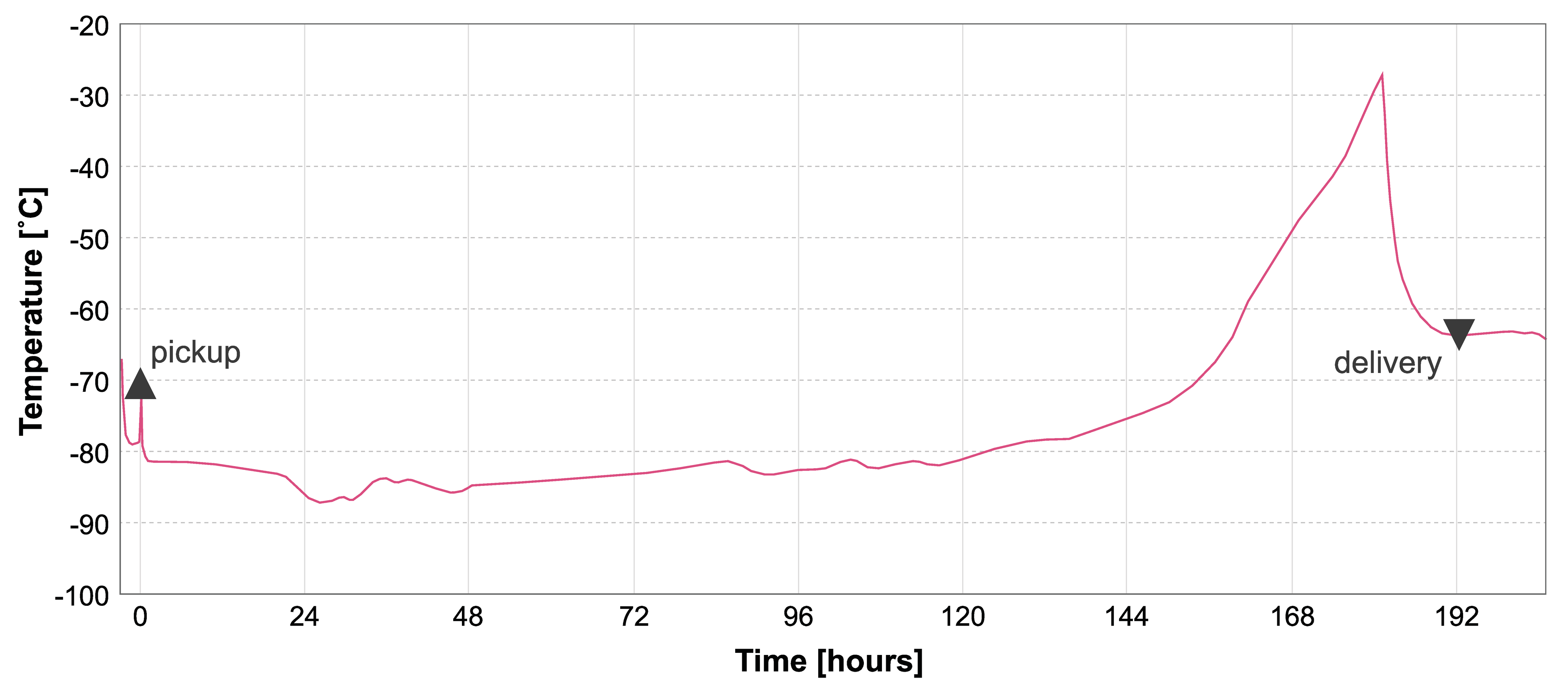

Supplement: S1 Fig — The figure details temperature monitoring for the shipment of HUMO samples and biocytometry kits from the centralized laboratory to the reference decentralized laboratory. Shipped with the expectation of maintaining -80°C on dry ice, a significant deviation was recorded by the temperature monitor between hours 144 and 192, with a peak at around -27°C. Given the temperature sensitivity of the HaCaT and HL-60 cell lines contained in the HUMO samples, this unexpected rise in temperature may have compromised their viability, potentially leading to cellular necrosis and impacting the integrity of the samples upon arrival. (TIF) [file pone.0314992.s003.tif]

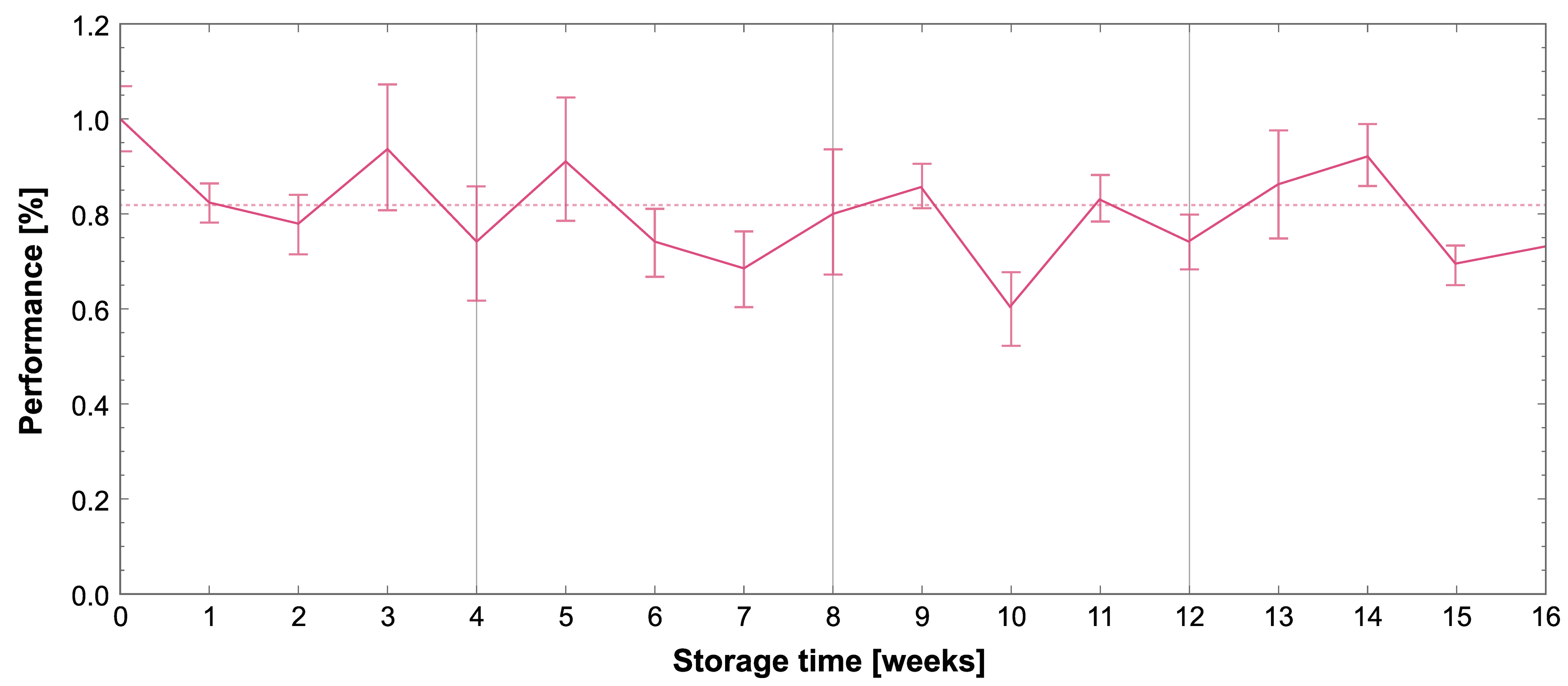

Supplement: S2 Fig — A longitudinal stability study of the bioparticles integral to the biocytometry kits demonstrated sustained performance, with no significant decline observed over a 16-week period when stored at -20°C, as depicted in S2 Fig. This stability indicates the temperature fluctuations recorded during the shipment, detailed in S1 Fig, did not compromise the efficacy of the biocytometry kits. Consequently, these findings validate the robustness of the study design and assure that the assay results remained unaffected by the temperature variances encountered in transit. (TIF) [file pone.0314992.s004.tif]

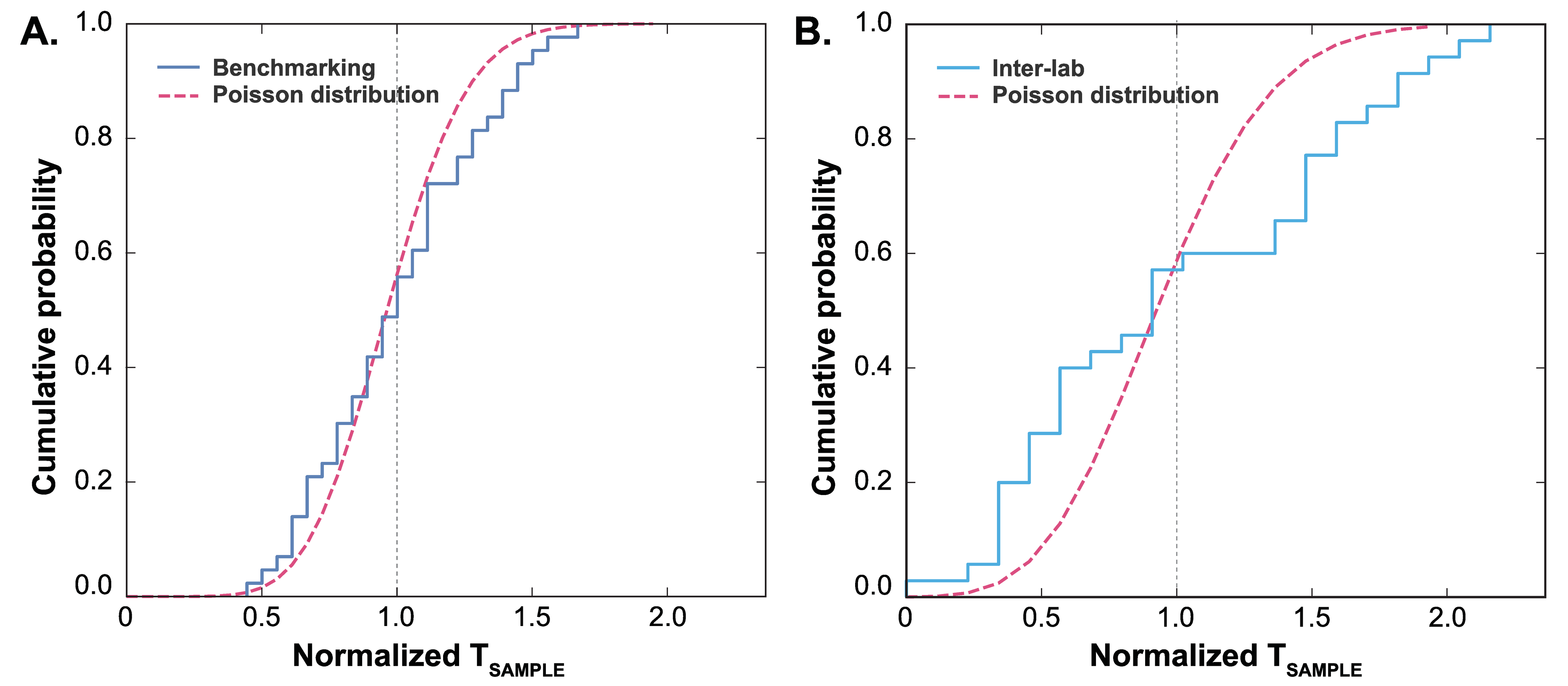

Supplement: S3 Fig — Figure presents cumulative distributions of normalized TSAMPLE values from high HUMO samples in (A) the benchmarking study and (B) the inter-lab study. Each TSAMPLE value was normalized by average TSAMPLE for its respective study, centering the distributions around 1. A) Observed CV was 31%, while the expected Poisson CV is 21%. B) Observed CV was 61%, while the expected Poisson CV was 34%. (TIF) [file pone.0314992.s005.tif]

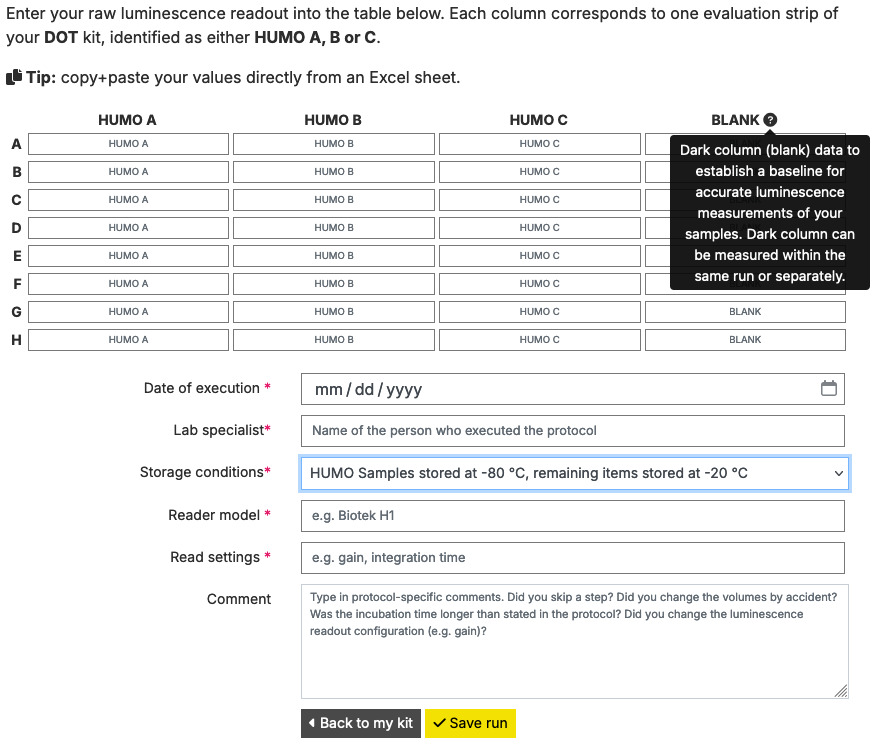

Supplement: S4 Fig — The submitted data were paired with the corresponding study participant and biocytometry kit. Note, biocytometry does not require any centralized data submission or processing system for future applications. Each user can independently calculate target cell estimates from raw luminescence data, using the provided conversion factor and formulas detailed in the Materials and Methods section. (TIF) [file pone.0314992.s006.tif]
