# Supplementary material for: Enhancing reproducibility in single cell research with biocytometry: An inter-laboratory study
Source: PLoS One. 2024 Dec 9;19(12):e0314992. doi: 10.1371/journal.pone.0314992 (PMC11627387; doi:10.1371/journal.pone.0314992)
Supplement: S2 File — (PDF) [file pone.0314992.s008.pdf]

De-hashing decentralized data

In order to ensure an unbiased analysis in the decentralized data collection phase, the HUMO samples were delivered to participants under a disguised nomenclature, obscuring the identity of each sample. The samples were labeled as HUMO A, HUMO B, and HUMO C instead of their actual types; low, high, and negative, respectively. Participants, therefore, engaged in blind analysis, unaware of the specific HUMO type they were analyzing. Upon receiving the data from the participants, a critical step of data de-hashing was performed. This involved reorganizing the submitted data to reflect the true identity of the samples, aligning them to their respective types - negative, low, and high HUMO. Through this meticulous process, the data was aptly restructured for subsequent analysis, while assuring an unbiased data collection and validation process.
