# Supplementary material for: Enhancing reproducibility in single cell research with biocytometry: An inter-laboratory study
Source: PLoS One. 2024 Dec 9;19(12):e0314992. doi: 10.1371/journal.pone.0314992 (PMC11627387; doi:10.1371/journal.pone.0314992)
Supplement: S3 File — (PDF) [file pone.0314992.s009.pdf]

Box charts of target estimates

Sample target estimates were split based on the respective sample type and assay cohort. Sample target estimates were then visualized in Figs 3C, 4B and 5C using a box chart for different users and assay cohorts. In the box chart, each box consists of a median marker, fences that represent quartiles, and whiskers that represent 5% and 95% quantiles. Quantiles were estimated using a mode method. Outliers were detected by a median based method. Individual well target estimates were visualized and compared to the target cell counts obtained by microscopy in Fig 2C.
