# Supplementary material for: Enhancing reproducibility in single cell research with biocytometry: An inter-laboratory study
Source: PLoS One. 2024 Dec 9;19(12):e0314992. doi: 10.1371/journal.pone.0314992 (PMC11627387; doi:10.1371/journal.pone.0314992)
Supplement: S4 File — (PDF) [file pone.0314992.s010.pdf]

Hypothesis testing

A hypothesis test was used to judge the ability of the technology to discriminate between sample types as follows. Assays were categorized into distinct cohorts based on assay and user metadata (e.g. distinct laboratory or user proficiency level). For each sample type, the sample means were consolidated across assays within a specific cohort. A Wilcoxon signed-rank test was subsequently executed, comparing the sample means of type i with those of type j within the given assay cohort. The p-value for this test was determined for every pertinent assay cohort and for each pairing of sample types. The outcomes of these tests were graphically represented in Figs 3A and 5A.
