# Supplementary material for: Enhancing reproducibility in single cell research with biocytometry: An inter-laboratory study
Source: PLoS One. 2024 Dec 9;19(12):e0314992. doi: 10.1371/journal.pone.0314992 (PMC11627387; doi:10.1371/journal.pone.0314992)
Supplement: S5 File — (PDF) [file pone.0314992.s011.pdf]

Histograms

SNRT values were further visualized using a smooth histogram in Fig 2B. The histogram was smoothed with a gaussian kernel.
